# Impact of Human Papillomavirus (HPV) on Male and Female Fertility

**DOI:** 10.3390/pathogens13121076

**Published:** 2024-12-07

**Authors:** Sara Chenafi-Adham, Oulfa Boussetta-Charfi, Sylvie Pillet, Thomas Bourlet

**Affiliations:** 1 Department of Infectious Agents and Hygiene, University Hospital of Saint-Etienne, 42023 Saint-Etienne, France; sarachenafi@gmail.com (S.C.-A.); boussetta.oulfa@gmail.com (O.B.-C.); sylvie.pillet@chu-st-etienne.fr (S.P.); 2 GIMAP Team 15, Centre International de Recherche en Infectiologie, INSERM U1111, CNRS, UMR5308, University of Saint-Etienne, University of Lyon, 42023 Saint-Etienne, France

**Keywords:** human papillomavirus, infertility, screening, vaccination

## Abstract

Human papillomaviruses (HPVs) are responsible for the majority of sexually transmitted infections (STIs), some of which are oncogenic and can cause oropharyngeal or genital cancers. The HPV prevalence at the genital level varies according to the population studied but is higher in the seminal fluid of men suffering from idiopathic infertility than in the general population. The involvement of HPV in male infertility is supported by several studies suggesting that this virus can affect sperm quality by altering sperm DNA integrity, motility, number, viability, and morphology, and by inducing the production of anti-sperm antibodies (ASAs). HPVs may also have an impact on female fertility, mainly by increasing the risk of miscarriage and premature delivery and by altering the implantation of endometrial trophoblastic cells. In addition, an association with vaginal bacterial dysbiosis, notably involving *Gardnerella vaginalis*, or co-infection with an STI agent, serves as an aggravating factor. The aim of this review is to present current data on the potential role of HPVs in male and female infertility, along with data on infertility prevention and treatment strategies and the impact of vaccination in this context.

## 1. Introduction

Infertility is defined as the inability to achieve pregnancy within one year of regular, unprotected sexual intercourse. It is estimated to affect between 8% and 30% of couples of childbearing age worldwide [1]. Male infertility alone accounts for 20 to 30% of cases and is associated with one or more other factors in around 30% of cases. An infectious cause in the genital tract is identified in around 15% of cases of male infertility [2], making it the third most frequent cause after idiopathic infertility and varicocele [3]. Consequently, sperm culture is included in the standard pre-treatment evaluation for assisted reproductive technologies (ARTs) to diagnose potential infections in the adnexal glands of the genital area [4,5].

In women, 20–60% of infertility cases are caused by sexually transmitted infection (STI) agents, which can in some cases lead to pelvic inflammation and tubal obstruction [6]. The most common bacterial pathogens responsible for infection in the female genital tract include mycoplasmas (*Mycoplasma hominis* and *Ureaplasma urealyticum)*, *Chlamydia trachomatis*, *Neisseria gonorrhoeae*, and *Gardnerella vaginalis*. Certain sexually transmitted viruses have also been described as associated with male and/or female infertility, namely human immunodeficiency virus (HIV), human papillomavirus (HPV), and, more recently, SARS-CoV-2, which seems to have a limited deleterious impact on sperm parameters over time [7,8]. Dysbiosis in the vaginal microbiome has also been suggested as a factor associated with infertility by numerous works and two recent reviews [9,10]. In particular, an increase in the genera *Atopobium*, *Aerococcus*, and *Bifidobacterium* and a decrease in the abundance of *Lactobacillus* and *Leuconostoc* have been reported [11].

The HPV virus belongs to the *Papillomaviridae* family and is responsible for the majority of STIs worldwide. It is estimated that around 80% of the sexually active population has been exposed to one or more genotypes of this virus and that around 12% of women are infected worldwide. However, the epidemiology differs according to gender; while genital prevalence is high in females during the sexually active years and declines thereafter, it remains stable and high in men throughout their lives [12]. The impact of HPV on infertility has been addressed by numerous studies, some of which report contradictory results, particularly in women. However, it should be emphasized that infertile women are almost twice as likely to have abnormal cervical cytology or high-grade cervical lesions caused by HPV infection [13]. HPV has, in fact, been isolated from endometrial lesions, suggesting a potential role of this virus in infertility in patients with pelvic endometriosis [14]. This observation is explained by the ability of HPV to migrate along the female genital tract and the endometrium and upward into the placenta via infected spermatozoa.

In men, the prevalence of HPV is higher than in women, as assessed by a recent meta-analysis reporting a global pooled prevalence of 31% (95% CI 27–35) for any HPV and 21% for HR-HPV [15]. To date, numerous studies have established a link between HPV infection and infertility. Firstly, the prevalence of HPV is higher in the semen of people suffering from idiopathic infertility than in the general population, suggesting a deleterious effect of this virus on seminal parameters and fertility [16]. This hypothesis is reinforced by the observation of the presence of the HPV genome and/or particles at the surface of spermatozoa and in the adnexal glands of the genital tract, leading to altered sperm parameters (reduced sperm count and motility in particular) [17,18]. Finally, there is the question of the influence of these viruses on procreation parameters, whether natural or medically assisted. In this sense, recent data obtained in vitro suggest an adverse impact of HPV on blastocysts and their multiplication as well as on early embryonic development [19].

The aim of this review is thus to present current data on the potential role of HPV in male and female infertility and to discuss prevention and treatment strategies as well as the impact of anti-HPV vaccination on infertility.

## 2. HPV and Female Infertility

Female infertility is idiopathic or unexplained in around 30% of cases [20]. There are several possible causes of female infertility, including ovulatory dysfunction, tubal patency, endometriosis, uterine anomalies, and STIs. To date, the direct impact of HPV on female fertility has not been clearly demonstrated, and few studies have been published on this topic. However, several studies have reported a high prevalence of high-grade cervical intraepithelial neoplasia (CIN2+) among infertile women, suggesting that HPV is a potential risk factor for infertility [21]. HPVs have also been isolated from endometrial lesions, which may suggest a role for this virus in the infertility of these patients affected by endometriosis [14]. Two other cohort studies, one conducted in Denmark and the other in Taiwan, produced contradictory results regarding the association between HPV and female infertility [22,23]. While in the Danish study, no association was found, in the Taiwanese one, a statistically significant correlation between HPV infection and infertility was described in the 26–35 age group compared with an uninfected cohort [23].

Focusing on HR-HPV genotypes, results remain controversial regarding a significant association with female infertility, even though a recent meta-analysis of eleven studies concluded that HPV infection represents a potential non-independent risk factor for female infertility [24]. Regarding the role of co-infections between HPV and other STI agents on infertility, Casari and colleagues suggested an aggravating role of *Gardnerella vaginalis* on the latter [25]. On the other hand, results remain contradictory for *Chlamydia trachomatis*, *Mycoplasma hominis*, and *Ureaplasma urealyticum* [26].

## 3. HPV and Male Infertility

A higher prevalence of HPV in the semen of men with idiopathic infertility has been reported in several studies [27,28]. A link between HPV and male infertility has been established by several authors describing the impairment of various seminal parameters, in particular sperm composition, pH, sperm motility, and chromosomal alterations (Table 1). In addition, oncogenic high-risk genotypes (hrHPV) 16, 31, 51, 42, and 56 have been described as predominantly associated with this infertility risk [29,30]. In particular, most hrHPV genotypes have been shown to alter sperm parameters such as progressive sperm motility and DNA fragmentation [18]. Infection with several of these genotypes also appears to be an aggravating factor [31]. From a pathophysiological point of view, it has been shown that HPV can Infect human spermatozoa, localizing in the equatorial region of the sperm head through interaction between the HPV capsid L1 protein and syndecan-1 [32].

## 4. Effect of HPV on Reproductive Parameters

As mentioned above, the HPV virus can bind to the surface of spermatozoa, thus potentially transmitting infection to the oocyte during fertilization [43]. Furthermore, it has been hypothesized that infected spermatozoa can also transmit infection to the cervix during fertilization and even reach the placenta via the hematogenous route by ascending infection, causing miscarriage and infertility in couples [44]. In infertile couples, Duan et al. reported a statistically significant correlation between reduced fertility and the HPV-positive status of the male partner [13].

Another theory concerning the transmission of viral DNA from sperm to embryo has been suggested in a mouse model by Mastora and colleagues [45]. Their study shows how the injection of HPV-infected sperm into the oocyte has a negative impact on fertilization outcome, implantation, and embryo development. The underlying hypothesis is that HPV interferes with the fusion stage between sperm and oocyte [27]. In the same vein, Perino et al. have shown a lower pregnancy rate and a higher abortion rate in pregnant women of HPV-positive couples compared with seronegative couples [46]. With regard to spontaneous abortion rates, a meta-analysis of 38 studies found no significant association with intra-couple HPV infection [47]. On the other hand, in a subgroup analysis of eight case-control studies, the authors demonstrated that infection was significantly associated with prematurity, early rupture of membranes, intrauterine growth retardation, low birth weight, and fetal death [47].

In brief, the precise mechanism by which HPV infection impacts obstetric fertility rates is not yet well understood, and the results remain controversial. Larger-scale studies are needed in this area.

In addition, it is also interesting to consider the potential indirect effect of HPV lesions management on infertility and pregnancy loss. Two reviews on this topic have warned about an association between conization or large loop excision of the transformation zone and an increased risk of subsequent perinatal mortality, other serious pregnancy outcomes, and preterm births [48,49].

## 5. Prevention and Management of HPV-Related Infertility

The prevention and management of HPV-related infertility can be based on several strategies. The first of these falls within the general framework of STI prevention and good sexual hygiene practices, which have been shown to significantly reduce HPV persistence in infected couples [50]. Secondly, given the data linking HPV with male infertility, the efficacy of HPV vaccination in preventing infertility merits consideration. Indeed, the association between male HPV infection and asthenozoospermia, along with the increased risk of pregnancy loss, supports the recommendation to extend HPV vaccination to adolescent males, thereby preventing HPV-related anogenital and oral cancers. Moreover, the vast majority of hrHPV genotypes involved in impaired sperm parameters are included in the 9-valent vaccine, namely 16, 18, 31, 33, 45, 52, and 58. A prospective study along these lines could be useful to confirm the impact of this primary prevention on couples’ reproductive health. On the other hand, recent research suggests that HPV vaccination is an effective treatment option for people who have already contracted the infection [51]. In a retrospective study of 99 men with HPV-infected semen who received the tetravalent vaccine, a higher viral clearance rate was evidenced, along with improved sperm motility and lower levels of anti-sperm antibodies [52]. In the same study, couples whose men were vaccinated had higher pregnancy and delivery rates.

Another way to manage young infertile couples undergoing MPA whose men are seminal HPV-infected would be to delay treatment for six months to facilitate the elimination of the infection [53]. From this point of view, further work is needed to assess more precisely the time required for viral clearance in the semen of these populations. Given that nitrogen is an excellent medium for preserving viruses, it could also be envisaged that HPV-positive semen samples should not be used for assisted reproduction or sperm banking. To this end, some authors advocate systematic HPV screening of sperm donors, in addition to the current microbiological work-up, on medico-economic grounds and in light of studies suggesting an association between HPV, infertility, and reproductive failure [54]. In particular, it is suggested that HPV may be tested in men’s sperm (from a couple undergoing MPA or from an anonymous donor) in the following cases: idiopathic infertility in couples, the discovery of asthenozoospermia, the presence of ASA, and infection [41].

Still, in the field of MPA, in an attempt to eliminate viral DNA, various sperm washing procedures have been proposed, such as the swim-up method, microfluidic sperm sorting, magnetic-activated cell sorting, and/or density gradient centrifugation [37]. The combination of the “swim-up” process and sperm treatment with heparinase III appears to be the most effective method [55].

Another option, under investigation, would be to prescribe substances with antioxidant properties in order to reduce oxidative stress, partly linked to HPV infection and the effects on DNA fragmentation [56].

In any case, the currently available data argue in favor of targeted HPV screening and the development of recommendations for the management of HPV-infected patients in the general population and/or in the context of MPA.

## 6. Conclusions

**Figure 1:**
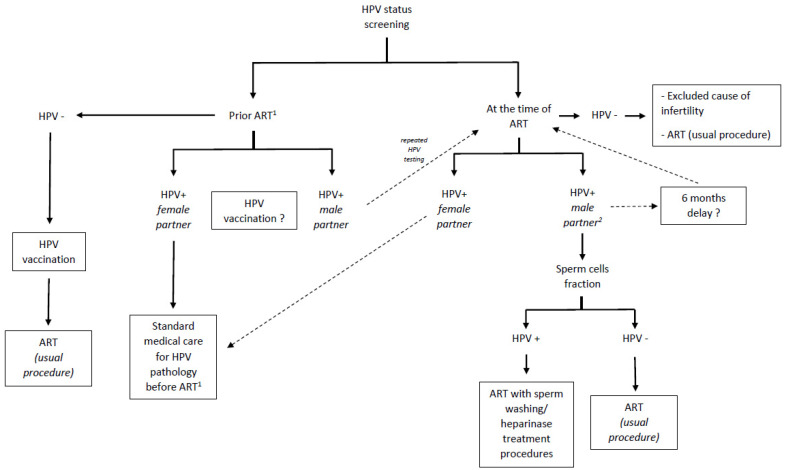
Proposal of an algorithm for HPV screening in the context of infertility diagnosis and medically assisted reproduction management. ART: assisted reproductive technology. ^1^ When possible, taking age into account for successful ART. ^2^ In whole semen.

It is now well-established that HPV plays a significant role in couples’ infertility, particularly through its association with sperm damage and reduced sperm quality, which impacts reproductive success rates. This connection underscores the importance of considering HPV screening as part of infertility treatments, although the cost–benefit analysis of such screening remains to be determined. Additionally, exploring strategies for natural virus clearance or using specific techniques to prepare seminal fractions in assisted reproductive technologies (ARTs) could be valuable. In this respect, we propose in Figure 1 a decision-making algorithm for screening in the context of medically assisted reproduction care. Finally, addressing infertility might also justify the use of preventive or therapeutic HPV vaccinations among infertile couples affected by HPV.

## 7. Future Directions

The question of the role of papillomaviruses on male or female infertility is part of the broader field of STI management and prevention. It also seems appropriate to include it in the broader strategy of invasive cervical cancer eradication, as proposed by the WHO with the 90-70-90 initiative based on vaccination coverage, screening, and treatment, respectively.

## Figures and Tables

**Table 1:** Main seminal parameters affected by HPV infection and potentially involved in male infertility.

Sperm Parameter	Effect of HPV Infection and Consequence(s)	Reference	Publication Category
Zinc	Decreased sperm production: sperm condensation defect	[33]	Book chapter
Citric acid	Decrease: increase in oxidative stress, deleterious effect on spermatozoa	[34]	Review
pH	Increased acidity: decreased seminal vesicle and ejaculatory duct activity	[35]	Original article
Sperm cells	Reduced mobility, altered vitality, number, and morphology	[36]	Meta-analysis
DNA	Increased fragmentation due to oxidative stress	[37]	Original article
	Increased apoptosis (HPV 16 and 31)	[38]	Original article
	Alteration of the p53 gene (HPV 16 and 18)	[39]	Original article
Seminal leukocytes	Increase	[40]	Original article
Pro-inflammatory cytokines IL-1, IL-6, and TNF-α	Increased by HPV and stimulating oxidative stress	[41]	Review
Anti-sperm antibodies (ASA)	Production due to degradation of spermatozoa by HPV: reduced sperm mobility	[42]	Original article
